# Prostate-specific antigen, sexual behavior, and sexually transmitted infections in US men 40–59 years old, 2001–2004: a cross – sectional study

**DOI:** 10.1186/1750-9378-2-19

**Published:** 2007-10-24

**Authors:** David M Werny, Mona Saraiya, Xiao Chen, Elizabeth A Platz

**Affiliations:** 1 Division of Cancer Prevention and Control, National Center for Chronic Disease Prevention and Health Promotion, Centers for Disease Control and Prevention, Atlanta, USA; 2 Department of Epidemiology, Johns Hopkins Bloomberg School of Public Health and the Brady Urological Institute, Johns Hopkins Medical Institutions, Baltimore, USA

## Abstract

**Background:**

Sexually transmitted infections (STIs) are hypothesized to play a role in the development of prostate cancer, perhaps due to inflammation-induced oncogenesis. We assessed in a nationally representative population of middle-aged men whether sexual behavior indicators for an increased risk of genital infection were associated with serum prostate-specific antigen (PSA) concentration, a marker of prostatic disease and inflammation.

**Results:**

The percentage of men between the ages of 40 and 59 with a PSA ≥ 4.0 ng/ml was 2.6% (95% confidence interval [CI], 1.8% – 3.8%). The percentage of men between the ages of 40 and 59 self-reporting a past diagnosis of genital warts or genital herpes, or a recent diagnosis of gonorrhea or chlamydia is estimated to be 7.3% (95% CI, 6.2% – 8.6%). Men self-reporting that they had had sex without using a condom in the past month had a lower PSA concentration and higher %fPSA than those who did not. There were no associations between any of the other sexual activity or laboratory measures and PSA or %fPSA.

**Conclusion:**

In this nationally representative sample of middle-aged American men, we did not find consistent evidence for an association between sexual behavior or a history of STIs and PSA levels. Therefore, sexual factors are unlikely to lead to falsely elevated PSA tests in this population. We cannot rule out the role of these factors in causing false positive PSA tests in subgroups of the population that have a higher prevalence of high-risk sexual behavior, and more protracted or recent exposures to these agents.

## Background

Prostate cancer is a significant source of morbidity and mortality around the world, yet its primary causes remain a mystery. Two recent meta-analyses of mostly case-control studies have suggested that a history of sexually transmitted infections (STIs), specifically human papillomavirus (HPV) and *Neisseria gonorrhoeae*, may be risk factors for prostate cancer. [1,2] Organisms infecting the prostate may induce either symptomatic or asymptomatic chronic prostatic inflammation, and several biological hypotheses have been proposed to support inflammation-induced prostate oncogenesis. [3] Currently the links between infectious agents, inflammation, and prostate cancer are poorly understood.

The prostate-specific antigen (PSA) test is commonly used both as a screening tool and as a part of the diagnostic workup to rule out prostate cancer. [4] Serum levels of PSA are influenced by factors apart from the presence of cancer and other prostatic conditions, including age, body mass index,[5] and perhaps race[6] Serum PSA can be divided into "free" and "complexed" portions depending on whether the circulating PSA protein is bound to protease inhibitors. The ratio of free PSA to total PSA, called the percent free PSA (%fPSA), is sometimes used as a reflex test for an elevated PSA result, with a lower %fPSA indicating an increased risk of prostate cancer[7]

PSA levels are known to increase during episodes of symptomatic bacterial prostatitis,[8] and they have been shown to vary by the histological extent of asymptomatic inflammation[9,10] One cross-sectional study of men at an STI clinic found an inverse association between age at first intercourse and mean PSA, as well as a positive association between higher titers of antibodies to *Chlamydia trachomatis *and PSA values. [11] High *C. trachomatis *antibody titers are likely caused by multiple repeat infections, or chronic, asymptomatic infections. A longitudinal study at two Baltimore STI clinics found that a subset of men who present acutely with STIs experience a sharp increase in PSA, suggesting that some non-ulcerating STIs may progress to infect the prostate, ultimately inducing inflammation. [12] These studies raise the question of a potential association between high-risk sexual behavior or STIs and increased PSA concentration. However, to date, no studies have estimated the effect of this association in a nationally representative population. In a population screened for prostate cancer, changes in PSA due to these factors may have additional importance, perhaps leading to false-positive PSA screening tests. We evaluated the association between sexual behavior, self-reported history of STIs, and PSA concentration from the 2001–2004 National Health and Nutrition Examination Survey (NHANES). Additionally, we examined the association of serological evidence of infection with *T. pallidum *(syphilis) or herpes simplex virus-2 (HSV-2) with serum PSA values. These STIs are themselves markers of high-risk sexual behavior, [13] but have not been shown to directly infect the prostate or to induce prostatic inflammation.

## Results

Characteristics and demographics of this study population have been described previously. [14] Of the 1513 interviewed men 40 to 59 years of age, 1456 (96.2%) participated in the physical examination. Of these 1456 men, 29 (2.0%) did not provide consent for PSA testing, refused to answer the consent question, or responded "don't know," 36 (2.5%) were considered ineligible because of other NHANES PSA exclusion criteria, an additional 65 (4.5%) men were missing information on either the consent question or at least 1 of the exclusion criteria, and 33 (2.3%) were eligible but had a missing PSA result, leaving a final study population of 1293 men (1293/1456, or 88.8% of all men between 40 and 59 years of age who participated in the physical examination). For the subgroup of men aged 40 to 49 similar percentages were excluded as above (a total of 84 excluded out of 831), leaving 747 for the analysis of this subgroup. Only 15 men were excluded from this analysis because of recent infection or inflammation of the prostate. The total number of respondents varied for each question; for all sexual behavior questions the percentage of missing and refusals varied from 0.5% to 12.7%. Men who were excluded from our analysis had the same median number of lifetime sexual partners, partners in the last year, and partners in the last month as those who were not excluded. Excluded men were not more likely to report a past STI than non-excluded men (p = 0.95). Race/ethnicity was not associated with PSA levels in this sub-section of the NHANES population.

The percentage of men between the ages of 40 and 59 with a PSA ≥ 4.0 ng/ml was 2.6% (95% confidence interval [CI], 1.8% – 3.8%). The percentages of seropositivity for *T. pallidum *and HSV-2 in men 40 to 49 years old are 1.6% (95% CI, 1.1% – 2.4%) and 17.5% (95% CI, 13.9% – 21.7%), respectively. The percentage of men between the ages of 40 and 59 self-reporting a past diagnosis of genital warts or genital herpes, or a recent diagnosis of gonorrhea or chlamydia is estimated at 7.3% (95% CI, 6.2% – 8.6%).

No trends were seen for predicted-margin geometric mean PSA by age at first intercourse, the number of female sex partners in the last year, or the number of female partners in the past month (Table 1). An irregular pattern of predicted-margin geometric mean PSA levels was noted over the number of lifetime sexual partners. Those men who had had sex without a condom in the past month had a lower predicted-margin geometric mean PSA than those who had not (0.76 ng/ml vs. 1.15 ng/ml, p = 0.031). No differences in predicted-margin geometric mean PSA were seen by self-reported history of an STI, by circumcision status, or by the antibody tests for HSV-2 and syphilis infection (Table 1 and Table 2).

**Table 1:** Sexual Behavior and PSA Concentrations among US Men (Age 40 – 59), NHANES 2001 – 2004

Variable	n	Predicted margin geometric mean PSA (ng/ml)	p-value	Predicted margin percent free PSA	p-value
Age at first intercourse (years)	1185				
≤ 15	400	0.80 (0.73 to 0.89)	Reference	30.3 (28.6 to 32.0)	Reference
16 – 18	476	0.81(0.76 to 0.87)	0.833	31.1 (29.5 to 32.6)	0.561
≥ 19	309	0.82 (0.75 to 0.90)	0.802	31.3 (29.6 to 32.9)	0.417
Number of lifetime sexual partners	1169				
0 – 3	268	0.83 (0.76 to 0.91)	Reference	31.0 (29.2 to 32.7)	Reference
4 – 5	181	0.94 (0.84 to 1.05)	0.072	31.3 (29.1 to 33.6)	0.820
6 – 10	268	0.73 (0.68 to 0.79)	0.069	31.4 (29.4 to 33.4)	0.783
11 – 20	193	0.91 (0.78 to 1.05)	0.345	29.2 (26.6 to 31.7)	0.317
20 +	259	0.73 (0.66 to 0.80)	0.072	31.4 (29.0 to 33.9)	0.767
Number of sexual partners in the past year	1190				
0	155	0.78 (0.68 to 0.88)	Reference	30.5 (28.1 to 32.9)	Reference
1	854	0.82 (0.78 to 0.87)	0.471	31.1 (30.0 to 32.1)	0.703
≥ 2	181	0.79 (0.69 to 0.90)	0.916	30.3 (27.4 to 33.1)	0.882
Number of sexual partners in the past 30 days*	194				
0	29	0.76 (0.58 to 1.00)	Reference	30.8 (25.6 to 36.1)	Reference
1	95	0.77 (0.65 to 0.92)	0.914	29.9 (27.1 to 32.6)	0.734
≥ 2	70	0.88 (0.70 to 1.11)	0.392	29.4 (24.1 to 34.7)	0.696
Have you had sex without a condom in the past 30 days?^†^	161				
Yes	119	0.76 (0.66 to 0.88)	Reference	30.8 (27.8 to 33.7)	Reference
No	42	1.15 (0.81 to 1.63)	0.029	24.7 (19.0 to 30.3)	0.031
Are you circumcised?	1211				
Yes	837	0.82 (0.77 to 0.86)	Reference	31.1 (30.0 to 32.2)	Reference
No	374	0.80 (0.72 to 0.90)	0.810	30.0 (27.3 to 32.7)	0.348
Self-reported history of STD infection	1182				
Yes	77	0.85 (0.74 to 0.98)	0.517	28.5 (25.2 to 32.0)	0.151
No	1105	0.81 (0.77 to 0.85)	Reference	31.1 (30.1 to 32.1)	Reference

*Question asked only of men who have had >1 lifetime sexual partner

^†^Question asked only of men who have had at least one sexual partner in the past 30 days and had >1 lifetime sexual partner

**Table 2:** PSA Concentration by Serum Antibody Testing among PSA Eligible Men (Age 40–49), NHANES 2001 – 2004

Variable	n	Predicted margin geometric mean PSA (ng/ml)	p-value	Predicted margin percent free PSA	p-value
Serum test for herpes simplex virus-2 exposure	737				
Positive*	176	0.70 (0.63 to 0.77)	0.231	30.2 (28.1 to 32.4)	0.228
Negative or Indeterminate^†^	561	0.74 (0.70 yo 0.79)	Reference	31.7 (30.4 to 33.0)	Reference
Serum test for *T. pallidum *exposure	737				
Current^‡ ^or remote^§ ^infection	27	0.84 (0.66 to 1.06)	0.253	28.7 (22.7 to 34.7)	0.398
No infection^¶^	710	0.73 (0.69 to 0.78)	Reference	31.5 (30.3 to 32.7)	Reference

* Serum has antibodies to HSV-2 gP-2 protein † Three results were indeterminate

‡ positive or indeterminate

• positive or indeterminate EIA test for *T. pallidum *antibodies, and rapid plasma reagin (RPR) ≥ 8

§positive EIA test, and 1 ≤ RPR ≤ 8 and a positive *T. pallidum*-particle agglutination (TP-PA)

¶ negative EIA test, or positive or indeterminate EIA test and RPR score 0–8 and negative TP-PA

Men who reported having sex without a condom in the past month had a higher %fPSA than those who did not (Table 1). No associations were noted for %fPSA by any of the other behavior variables. No significant differences in %fPSA were seen by past HSV-2 or syphilis exposure as determined from serum assays.

## Discussion

Our predominantly null results do not support a link between sexual behavior or STIs and PSA concentrations in this nationally representative sample of US men 40–59 years of age, with the exception of recent sex and condom use. Therefore, sexual factors are unlikely to lead to falsely elevated PSA tests in this population.

We found a statistically significant lower PSA concentration and a higher %fPSA in men who had had sex without a condom in the last month. We are unsure as to why men who have had recent unprotected sex would have a lower PSA than those that have not. It should be noted that these analyses were limited by small numbers of men, and the possibility remains that this statistically significant result is a chance finding.

There are several reasons for our predominantly null results in this analysis. The lack of association between PSA and the number of lifetime sexual partners or the number of sexual partners in the last year may be because past STIs never infected the prostate, or infected the prostate but were treated and thus no longer induce inflammation. Additionally, men may have been infected asymptomatically, and now report never having had an STI. The antibody data collected in the NHANES do not allow us to consider the precise time of infection or current infection status of those testing positive for exposure to HSV-2 or syphilis. These 2 organisms are not believed to infect the prostate, and hence are unlikely to cause prostatic inflammation themselves; we considered these agents as surrogates for sexual behaviors and the opportunity for the acquisition of other STIs that do infect the prostate. Of the men self-reporting a past diagnosis of an STI (n = 77), most reported either a genital warts or genital herpes diagnosis (n = 71), and only 6 men reported a diagnosis of gonorrhea or chlamydia. Given that the HPV subtypes responsible for genital warts are rarely oncogenic, and that HSV-2 is not known to infect the prostate, we likely misclassified some of the men regarding likelihood of prostatic inflammation. Furthermore, men were asked to report gonorrhea and chlamydia diagnoses only within the past 12 months. Therefore, men who had had these infections in their young adulthood were combined with men who had never been infected with *N. gonorrhea *or *C. trachomatis*. All of these possible sources of misclassification, which would have biased our results toward the null, may have limited our ability to detect associations of a small magnitude between sexual behavior and PSA concentrations.

The PSA component of the NHANES excluded any man self-reporting current prostatic inflammation or infection, a well-established elevator of serum PSA. [15] This criterion likely excluded men with symptomatic prostatitis from our study, but we likely included men with asymptomatic chronic prostatitis because they are usually unaware of their condition. Men with mild prostatitis that has not been diagnosed by a doctor are also possibly included in this analysis. Because men with symptomatic prostatitis were excluded we may have underestimated the associations of STIs and sexual behavior with PSA concentration. We do not believe this is a substantial underestimation, because in the antibiotic era it is likely that men undergo curative treatment for a symptomatic STI before it infects the prostate. Men who do not seek treatment for a symptomatic STI likely make up a small portion of the general US population.

It is likely that some proportion of the men in this analysis had occult prostate cancer, which might have influenced PSA concentration. However, the prevalence of cancer in our study population is likely to be low, because all of our participants were younger than 60. [16] The majority of our analysis was restricted to men between the ages of 40 and 59. We do not feel this negatively impacts our analysis, and it may be a positive aspect of it. The inflammatory events that predispose men to later developprostate cancer are likely to have occurred many years prior to the diagnosis. The chronic inflammatory state induced by these events may be detectable as elevated PSA levels during these pre-diagnosis years, the time period where our cross-sectional analysis was conducted. One of the reasons our analysisdid not find a positive association with PSA could be that the infections precipitating the chronic inflammatory state thatleads toprostate cancer occurmostly while men are older than the men in our study. Prostate volume is also associated with serum PSA, [17] and we were unable to control for it in our analysis. The extent to which these 2 factors are associated with sexual behavior or STIs is unknown, and it is possible that they are confounders in this analysis.

The NHANES is not an ideal population in which to study the association between PSA values and a specific STI for a number of reasons. For one, the population is selected and weighted to represent the US population as a whole, not the high-risk sub-population you would expect to find at an STI clinic. This limited the number of STIs captured in this analysis, and forced us to collapse information into binary variables, in the process losing information on the association between PSA values and specific STIs. Another methodological limitation is that the NHANES is a cross-sectional study, and as such we have no information on the participants' numbers of previous STIs, in the cases where there was more than one episode of infection. This would have been valuable information, as men with multiple previous infections are more likely to have had prostate involvement. For these reasons we cannot rule out the role of sexual factors in causing false positive PSA tests in subgroups of the population that have a higher prevalence of high-risk sexual behavior, and more protracted or recent exposures to these agents.

However, the NHANES population-based sampling is also a strength of this analysis, allowing us to extrapolate these results to the male US population. Nationally representative analyses are ideal for studies of population-wide screening tools such as the PSA test, because they reveal the magnitude of potential associations over the entire population that uses the test. This study suggests that the prevalence of sexually transmitted infections of the prostate in the overall US population is relatively low, leading one to conclude that they do not greatly affect the accuracy of the PSA test at this population level.

Self-reported data on sexual behavior are susceptible to a number of response biases. [18] The sexual behavior data used in this analysis were collected using a computer-based system that allowed respondents complete privacy. Because of this enhanced privacy, computer-assisted self-interviewing methods are believed to be least susceptible to the motivational biases encountered commonly in these assessments. However, over-reporting of low frequency estimates and under-reporting of high frequency estimates is a common source of misclassification and may have influenced these results toward the null. [19]

## Conclusion

We report no association between PSA levels and sexual behavior or history of STIs in middle-aged American men. In this population, sexual behavior or a history of STIs is unlikely to affect PSA levels on a population-scale. We cannot rule out the role of these factors in causing false positive PSA tests in subgroups of the population that have a higher prevalence of high-risk sexual behavior, and more protracted or recent exposures to these agents.

## Methods

The NHANES is a nationally representative weighted sample of the US population. It consists of three parts: a questionnaire, a medical examination, and a blood draw. A description of the NHANES and the PSA testing component has been published previously. [14] Serum PSA and %fPSA was evaluated in all men aged 40 and older. Men were excluded from the NHANES PSA examination if they refused the PSA test, or if they self-reported a current infection or inflammatory condition of the prostate, having a digital rectal examination within the last 7 days, having a cystoscopy or biopsy within the last month, or having a past diagnosis of prostate cancer.

We categorized self-reported race/ethnicity as non-Hispanic white, non-Hispanic black, Mexican American, and other (other Hispanic men and all others). Participants were asked about their age at first intercourse, and their number of female sexual partners in the past year and over their lifetime. Those with more than 1 sexual partner in the last year were queried on the number of sexual partners in the last 30 days. Those with at least 1 sexual partner in the past 30 days were asked whether they had had sex without a condom in the last 30 days. Men were also asked if they had been told by a doctor or health professional in the last 12 months that they had chlamydia or gonorrhea, or if they had ever been told by a doctor or health professional they had genital herpes or genital warts. Because of a low number of affirmative responses (n = 77; 71 men reported genital warts or genital herpes, 6 reported a chlamydia or gonorrhea infection in the past 12 months), we combined these questions to form a binary variable for any of these 4 self-reported STIs. Men also self-reported whether they are circumcised. Sexual history and behavior questions were not asked of participants who were older than 59.

The NHANES syphilis testing component consists of three tests that determine history of exposure to *T. pallidum*: an enzyme immunoassay for IgG antibodies to *Treponema *antigens, a rapid plasma reagin assay for cell damage due to possible *Treponema *infection, and a particle agglutination test for IgG antibodies to *Treponema *to confirm indeterminate results from the other 2 assays. [20] Those men scoring 8 or higher on the rapid plasma reagin assay were considered to have a recent infection with *T. pallidum*, while those scoring less than 8 were classified as distantly infected. Recent and distant infections were combined because of the low number of *Treponema-*positive men overall (27/737 = 3.7%). The HSV-2 NHANES testing consists of an immunodot assay for the HSV-2-specific glycoprotein gG-2. [21] Of the 737 men tested, 176, or 23.9%, tested positive. Positive results are confirmed with a monoclonal antibody inhibition assay. Antibody tests for syphilis and HSV-2 were not conducted for participants older than 49.

Predicted-margin geometric mean PSA concentration and predicted-margin %fPSA, controlling for age and race/ethnicity, were calculated by sexual behaviors and STI status. A predicted margin for a given group is the average predicted value for a population, assuming every member of the population is in that group. [22] PSA values were log-transformed to improve the normality of the distribution. The %free values did not need to be log-transformed for normality. Accordingly, geometric mean PSA values and arithmetic mean %fPSA are reported in these analyses.

## Abbreviations

HPV – Human papillomavirus

HSV-2 – Herpes simplex virus-2

fPSA – Free PSA

NHANES – National Health and Nutrition Examination Survey

PSA – Prostate-specific antigen

STI – Sexually transmitted infection

## Competing interests

The author(s) declare that they have no competing interests.

## Authors' contributions

DW wrote and modified drafts, and carried out a portion of the statistical analysis. MS and EP provided guidance and input during the writing process, and both provided important intellectual content. XC contributed importantly to the statistical analysis.
